# Experimental evidence of nitrogen control on *p*CO_2_ in phosphorus-enriched humic and clear coastal lagoon waters

**DOI:** 10.3389/fmicb.2013.00011

**Published:** 2013-02-06

**Authors:** Roberta B. Peixoto, Humberto Marotta, Alex Enrich-Prast

**Affiliations:** ^1^Laboratory of Biogeochemistry, Department of Ecology, Institute of Biology, Universidade Federal do Rio de JaneiroRio de Janeiro, Brazil; ^2^Sedimentary and Environmental Processes Laboratory (LAPSA/UFF), Department of Geography, Institute of Geosciences, Universidade Federal FluminenseNiterói, Brazil

**Keywords:** eutrophication, *p*CO_2_, nitrogen, humic coastal lagoons, clear water coastal lagoons

## Abstract

Natural and human-induced controls on carbon dioxide (CO_2_) in tropical waters may be very dynamic (over time and among or within ecosystems) considering the potential role of warmer temperatures intensifying metabolic responses and playing a direct role on the balance between photosynthesis and respiration. The high magnitude of biological processes at low latitudes following eutrophication by nitrogen (N) and phosphorus (P) inputs into coastal lagoons waters may be a relevant component of the carbon cycle, showing controls on partial pressure of CO_2_ (*p*CO_2_) that are still poorly understood. Here we assessed the strength of N control on *p*CO_2_ in P-enriched humic and clear coastal lagoons waters, using four experimental treatments in microcosms: control (no additional nutrients) and three levels of N additions coupled to P enrichments. In humic coastal lagoons waters, a persistent CO_2_ supersaturation was reported in controls and all nutrient-enriched treatments, ranging from 24- to 4-fold the atmospheric equilibrium value. However, both humic and clear coastal lagoons waters only showed significant decreases in *p*CO_2_ in relation to the controlled microcosms in the two treatments with higher N addition levels. Additionally, clear coastal lagoons water microcosms showed a shift from CO_2_ sources to CO_2_ sinks, in relation to the atmosphere. Only in the two more N-enriched treatments did *p*CO_2_ substantially decrease, from 650 µatm in controls and less N-enriched treatments to 10 µatm in more N-enriched microcosms. Humic substrates and N inputs can modulate *p*CO_2_ even in P-enriched coastal lagoons waters, thereby being important drivers on CO_2_ outgassing from inland waters.

## INTRODUCTION

Carbon dioxide (CO_2_) is one of most important greenhouse gas in terms of global warming (IPCC, 2007; Royer et al., 2007; Solomon et al., 2010). The terrestrial biomass represents a relevant global stock of carbon (C), which is removed from the atmosphere by primary production (Gough et al., 2008). However, a significant part of this terrestrial organic matter leaches into aquatic ecosystems, where it may be buried in bottom sediments (Downing et al., 2008) or remineralized to CO_2_ by aquatic biological decomposition (Aufdenkampe et al., 2011). In the watershed, most natural inland waters are relatively small, but their wide geographic distribution, high abundance, and common location at low altitudes make them a typical fate for the water inflow from broad areas, playing a crucial role on the global C cycle (Cole et al., 2007).

Coastal lagoons are ecosystems often altered by the human land use (Marotta et al., 2010b), which show intense C fluxes (Duarte et al., 2008; Marotta et al., 2010b) The terrestrial inputs from leaching and groundwaters enhance CO_2_ in lakes by the contribution of inorganic C (Raymond et al., 1997; Marotta et al., 2010b), or organic substrates supporting the aquatic respiration (del Giorgio et al., 1997; Jonsson et al., 2003). Photosynthesis and respiration are the major metabolic pathways determining whether what level organic matter is produced and destroyed (Cole et al., 2000). Indeed, high terrestrial organic inputs may explain the positive general trend reported between dissolved organic carbon (DOC) and the partial pressure of CO_2_ (*p*CO_2_) in lake waters (Jonsson et al., 2003). Several studies have showed positive relationships in DOC and *p*CO_2_ in lakes in high latitudes and even globally (Sobek et al., 2005) supporting the idea that lakes are an important source of CO_2_ globally (Cole et al., 1994, 2007; Duarte and Prairie, 2005; Tranvik et al., 2009). Mean areal rates of CO_2_ evasion from lakes are higher at low latitudes, probably by the potential positive effect of warmer conditions on the organic decomposition (Marotta et al., 2009; Kosten et al., 2010). In this way, the degradation of organic matter to CO_2_ by bacteria shows important fluxes in the carbon cycling in natural aquatic ecosystems (Azam, 1998).

Additionally, the expansion of the human activities has intensified substantially the nitrogen (N) and phosphorus (P) input into ecosystems, often resulting in the eutrophication of natural waters (Vitousek and Mooney, 1997). These nutrients regulate aquatic primary production and respiration (Cole et al., 2000; Biddanda et al., 2001). Highly productive waters due to external inputs of inorganic nutrients tend to be net autotrophic, acting as a net sink for CO_2_ (Duarte and Agusti, 1998), while, those waters are highly enriched in organic substrates may show persistent CO_2_ supersaturation (Carpenter et al., 2001; Marotta et al., 2012).

Despite consistent evidences supporting the role of the limitation by either P (Schindler et al., 2008) or N (Camacho et al., 2003), N and P co-limitation may be also crucial on the biological metabolism in natural waters (Conley et al., 2009; Paerl, 2009). The biological N fixation can contribute to reduce the role of N inputs to stimulate biological activity in P-enriched waters, although more evidences is still needed for a better understanding on N limitation in coastal lagoons waters, where P is commonly enriched by domestic discharges.

In this study, we assessed the short-term effect of N additions on *p*CO_2_ in P-enriched humic and clear coastal lagoons waters. We tested the hypotheses that lake *p*CO_2_ is controlled by N availability in P-enriched waters.

## MATERIALS AND METHODS

### STUDY AREA

The experiment was conducted using surface waters from two tropical coastal lagoons situated at the same conservation area (Restinga de Jurubatiba National Park) in the north of Rio de Janeiro State (Brazil). Both coastal lagoons are elongated, with their main axis perpendicular to the shoreline (maximum depth <4.5 m; area <6.5 km^2^), oligotrophic (nutrients and chlorophyll *a* in the **Table 1**) and relatively close to each other (distant 6.8 km). The mean daily temperature in this area ranges from 20.7°C in July to 26.2°C in February. Despite high inter-annual variability, the minimum and maximum monthly rainfall are typically observed in August (38 mm) and December (182 mm; INMET, 1992). The tropical climate reflects in warm coastal lagoons waters (>20°C).

**Table 1:** Nutrients, chlorophyll *a*, color, DOC, Color:DOC ratio, salinity (PSU – practical salinity unity), and pH in surface waters of Carapebus and Comprida coastal lagoons used in experimental microcosms. Values are means and units of each variable are described below.

Lagoon	Total N (µmol l^-^^1^)	Total P (µmol l^-1^)	Chlorophyll *a* (µg l^-1^)	Color (430 nm)	DOC (mg l^-1^)	Color:DOC ratio (abs at 430 nm:mg l^-1^)	Salinity (PSU)	pH
Carapebus	45.3	1.0	13.8	0.014	9.84	0.0014	5.1	7.84
Comprida	58.1	0.4	2.5	0.102	17.43	0.0058	0.1	5.66

Carapebus coastal lagoon (22°13′21.29′′S and 41°36′53.22′′W) has clear waters, while Comprida coastal lagoon (22°16′44.55′′ S and 41°39′24.76′′W) has highly humic and dark waters. The dark color and high Color:DOC ratio in coastal lagoons waters of this region commonly reflects a higher contribution of terrestrial organic compounds from Restinga vegetation (Marotta et al., 2010a).

### EXPERIMENTAL DESIGN

Surface waters from both coastal lagoons were incubated in open-air 3.0-l glass bottles (microcosms) directly exposed to sunlight and other weather changes next to the studied coastal lagoons in June 2003. Solar incidence was the same for all microcosms, as they were placed close to each other, representing common light conditions for surface waters near to the interface with the atmosphere. However, the light attenuation indicated by Secchi depth at the sampling time was different between both, almost threefold above in Comprida lagoon than in Carapebus lagoon (1.6 and 0.5 m, respectively). No rainfall had been recorded during the incubations and the water temperature inside the microcosms varied between 25 and 30°C during the experiment. The evaporation contributed to negligence water level reduction inside microcosms, which was compensated by adding filtered waters from the same lake during the experiment.

The experiment was carried out over 15 days in highly P-enriched treatments in which different amounts of N were added, and the control (i.e., no N addition) per coastal lagoon. Three replicates were used in each experimental treatment and the control totalizing 24 microcosms. 1.4 μM of P as KH_2_PO_4_ and K_2_HPO_4_ (1:1 mass ratio to attenuate changes in pH) and 2.8, 28, and 120 μM of N as KNO_3_ were daily added to +N+P, ++N+P and +++N+P treatments, respectively. Nutrients were carefully added during the morning. Total additions were 20 μM P and 40, 400, and 1600 μM N in +N+P, ++N+P and +++N+P treatments, respectively, during the experiment. These concentrations and the corresponding N:P ratio were chosen to simulate the nutrient levels typically observed in urban coastal lagoons at the same region outside the Restinga de Jurubatiba National Park. The control microcosms showed only the low nutrient levels observed in both environments (0.4 and 0.9 μM P and 58.1 and 45.2 μM N, respectively in Carapebus and Comprida lagoons). All measurements were performed by the end of the experimental time (day 15).

### ANALYTICAL METHODS

pH was measured with a precision of 0.01 pH units using a Analion PM 608 pH meter and the total alkalinity following the Gran’s titration (APHA, 1992). Temperature and salinity were measured with a calibrated Thermosalinometer YSI-30. CO_2_ concentrations in waters were determined using the pH-alkalinity method (Mackereth et al., 1978) with appropriate corrections for temperature, altitude, and ionic strength as Cole et al. (1994). *p*CO_2_ was calculated from Henry’s law with appropriate corrections for temperature and salinity (Cole and Caraco, 1998) as in Marotta et al. (2010a).

Water samples for total P and N analyses were previously frozen. Total P concentrations were measured by the molybdenum blue method with pre-digestion and total N concentrations by the sum of Kjeldahl N and NOx forms (APHA, 1992). Chlorophyll *a* concentrations (a proxy for phytoplankton biomass) in water samples filtered through Whatman GF/F filters (0.7 μm pore size) were extracted with ethanol in the dark for 24 h before fluorimetric determination, using an excitation wavelength of 433 nm and an emission wavelength of 673 nm (Varian Eclipse). Total suspended solids (TSS) were analyzed by the difference of weight before and after filtering and drying GF/F filters. Water samples filtered in these Whatman GF/F filters were also analyzed for color at 430 nm (Strome and Miller, 1978) using a Beckman DU 80 spectrophotometer (Fullerton, CA, USA) in a 1-cm quartz cuvette, and acidified to pH < 2.0 to determine DOC by the high-temperature catalytic oxidation method using a TOC-5000 Shimadzu Analyzer. The bacterial production was estimated from the rate of incorporation of ^3^H-leucine (Smith and Azam, 1992), assuming a ^3^H-leucine dilution factor of 2 and a carbon:protein ratio of 0.86 (Wetzel and Likens, 1991). A volume of 1.3 ml of water from the microcosms and placed in an eppendorf (1.5 ml). In all tubes, rejoinders were added 20 μl of 3H-leucine (5× diluted standard solution, 159 mCi mol^-1^, Amersham), reaching a final concentration of 20 nM and incubated for 45 min in the dark. After the incubation period, were added in rejoinders, 90 μl of 100% trichloroacetic acid (TCA) stopping and starting the reaction extraction. Each tube was washed sequentially with 5% TCA and 80% ethanol and 500 μl of scintillation cocktail (Aquasol and Dupont) was added to each tube and the radioactivity measured in a liquid scintillator. Bacterial production was calculated by assuming a dilution factor of intracellular leucine equal to 2, and a protein rate of carbon equal to 0.86 (Wetzel and Likens, 1991).

### STATISTICAL ANALYSIS

The data were log-transformed (except pH) to meet the assumptions of parametric tests, including significant Gaussian distribution (Kolmogorov–Smirnov, *p* > 0.05) and homogeneity of variances (Bartlett, *p* > 0.05). Hence, differences among experimental treatments and the control were tested with one-way ANOVA (significance *p* < 0.05) followed by the Tukey–Kramer *post hoc* test (significance *p* < 0.05). All statistics were performed using GraphPad Prism 5.01 for Windows.

## RESULTS

**FIGURE 1:**
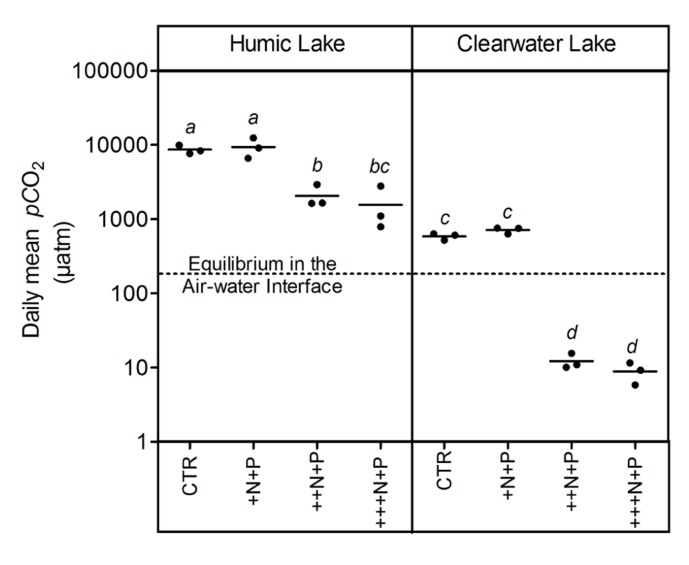
**Daily mean *p*CO_**2**_ after different N additions for humic and clear waters, respectively from Lake Comprida and Lake Carapebus at the last day of the experiment**. Each solid circle indicates one microcosm and the horizontal line the average. The four treatments are control (no additional nutrients) and three N levels (+N, ++N, and +++N, respectively 40, 400, and 1600 μM N – KNO_3_) +P addition (+P 20 μM P – KH_2_PO_4_ and K_2_HPO_4_). No significant differences among treatments and lake waters were represented by equal lower case letters (Tukey–Kramer, *p* > 0.05). The dashed line represents the *p*CO_2_ value at equilibrium with the overlying atmosphere (380 μatm). Note that values are in log scale.

Humic water microcosms from Comprida coastal lagoon showed average *p*CO_2_ values 10-fold higher than clear waters from Carapebus coastal lagoon in the controls and treatments +N+P, ++N+P, +++N+P (Tukey–Kramer, *p* < 0.05; **Figure 1**). A comparison between control and the less N-enriched treatment (+N+P) showed no significant difference in *p*CO_2_ among them, both in clear and humic waters (one-way ANOVA, *p* > 0.05; **Figure 1**). In contrast, these *p*CO_2_ values in control and +N+P treatments were significantly higher (Tukey–Kramer, *p* < 0.05; **Figure 1**) than those respective humic or clear water with higher N-additions (++N+P and +++N+P), which were also not significantly different between them (one-way ANOVA, *p* > 0.05; **Figure 1**). CO_2_ supersaturation was persistent in all humic treatments but not in clear water microcosms. The clear water microcosms presented a shift from being a source of CO_2_ in the controls and +N+P treatment to becoming a sink in ++N+P and +++N+P treatments in relation to the atmosphere (**Figure 1**).

**FIGURE 2:**
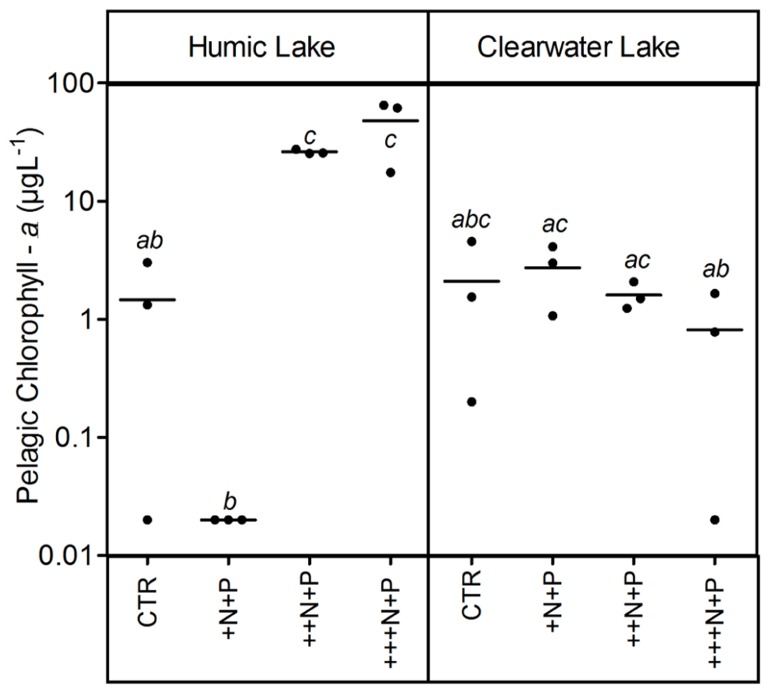
**Pelagic chlorophyll *a* after different N addition for humic and clear waters**. Note that values are in log scale. Legend as described in **Figure 1**.

**FIGURE 3:**
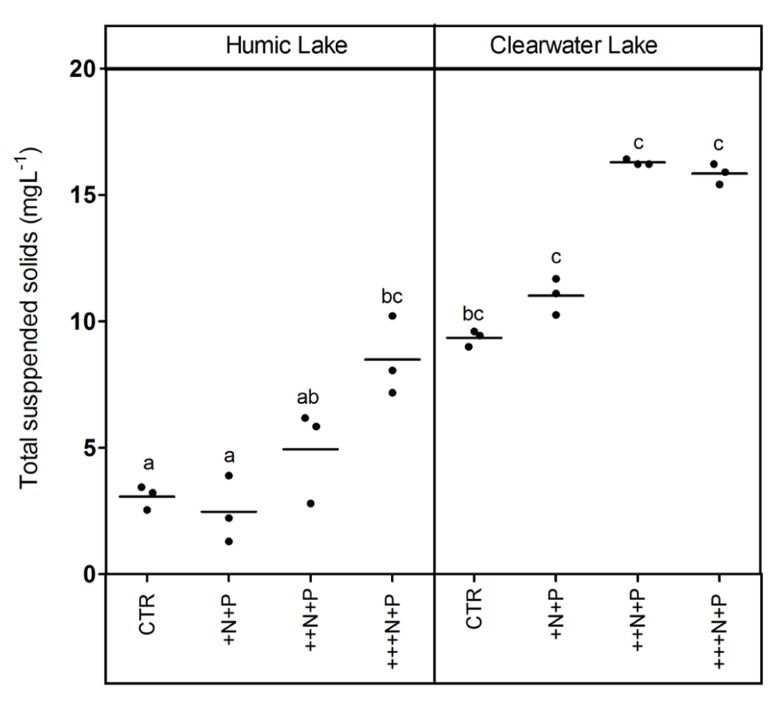
**Total suspended solids (TSS) after different N addition for humic and clear waters**. Legend as described in **Figure 1**.

The humic water microcosms also showed no significant difference (one-way ANOVA, *p* > 0.05) for pelagic chlorophyll *a* and TSS comparing controls and +N+P. Additionally, these less N-enriched humic treatments (control and +N+P) showed chlorophyll *a* significantly lower than ++N+P or +++N+P, and TSS significantly lower only than +++N+P (Tukey–Kramer, *p* < 0.05; **Figures 2** and **3**). However, the clear water microcosms showed no differences between treatments when chlorophyll *a* and TSS were all compared (one-way ANOVA, *p* > 0.05; **Figures 2** and **3**, respectively). Farther, humic water microcosms did not show any periphytic biomass on the microcosm wall, while a thick green periphytic biomass (non-pelagic microalgae) was observed at the edges of the ++N+P and +++N+P treatments microcosms.

**FIGURE 4:**
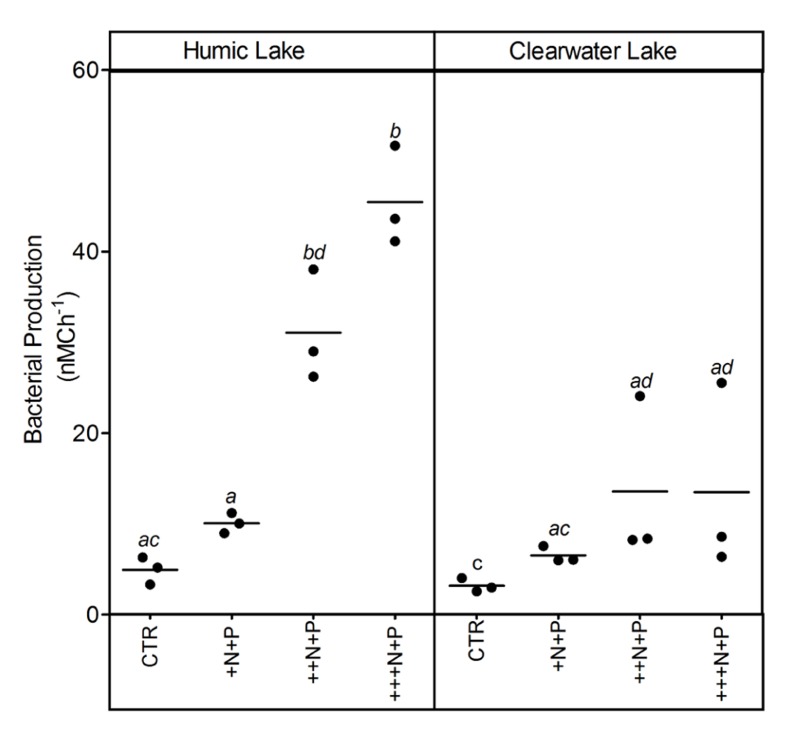
**Bacterial production after different N addition for humic and clear waters**. Legend as described in **Figure 1**.

Bacterial production increased with the amount of N added in both humic and clear water lake microcosms. However, this increase was significantly higher and more evident at the ++N+P and +++N+P humic lake water microcosms (Tukey–Kramer, *p* < 0.05; **Figure 4**).

## DISCUSSION

Overall, the humic waters from Comprida coastal lagoon showed a persistent CO_2_ supersaturation reaching higher *p*CO_2_ values than the controls or respective treatments with clear waters from Carapebus coastal lagoon. The humic nature of waters in Comprida coastal lagoon reflects the terrestrial DOC supply to heterotrophic bacteria in these ecosystems (Farjalla et al., 2009). Allochthonous organic resources contribute to high respiration rates and subsequently *p*CO_2_ within most lake waters (Duarte and Prairie, 2005; Cole et al., 2007). These results support the conclusion that, in that humic coastal lagoons waters have higher *p*CO_2_ values than the clear coastal lagoons, probably due to the more intense respiration of organic substrates (Marotta et al., 2010a).

Furthermore, P-enriched microcosms with higher N additions showed higher bacterial production rates and algal biomass (pelagic or periphytic chlorophyll *a*), suggesting that the N supply might limit the heterotrophic and autotrophic metabolic activity in P-enriched tropical coastal lagoon. Despite N_2_ fixation may be sufficient to allow biomass to continue to be produced even with extreme reductions in N inputs into lakes (Schindler et al., 2008; Smith and Schindler, 2009), our experimental evidences confirm that N might be a relevant control on eutrophication in coastal waters as previously pointed out (Conley et al., 2009; Paerl, 2009).

The CO_2_ balance was determined by higher N inputs, as higher N treatments showed strong net decreases in *p*CO_2_, supporting the potential role of aquatic primary producers on CO_2_ uptake (Carignan et al., 2000). Both heterotrophs and autotrophs are stimulated by the nutrient additions (Biddanda et al., 2001), although the net autotrophy may be favored in the balance, a general trend often reported for natural waters (Duarte and Agusti, 1998). Our results contrasted with the persistence of CO_2_ supersaturation in highly organic-enriched waters from whole-lake (Cole et al., 2000) or mesocosm studies (Marotta et al., 2012) also assessing the effects of experimental nutrient additions. One plausible explanation for this discrepancy would be the absence of the bottom sediment as an additional source of organic substrates to CO_2_ production within the microcosms.

Increases in the phytoplankton biomass (pelagic chlorophyll *a*) contributed to net CO_2_ decreases in highly N- and P-enriched microcosms with humic waters of the Comprida coastal lagoon, but not in those with clear waters of the Carapebus coastal lagoon, where no significant differences in pelagic chlorophyll *a* were reported among all experimental treatments or controls. Indeed, the CO_2_ decrease observed in more N- and P-enriched clear water microcosms was mainly related to the presence of periphyton biomass on the walls, which was absent in the humic water microcosms likely due to light attenuation to primary production in their dark waters (Thomaz et al., 2001). In humic waters, TSS increase might be related to the phytoplankton growth, as the *p*CO_2_ decreased without any periphyton growth on the microcosm walls. On the other hand, higher concentrations of non-algal solids in suspension (TSS not related to changes in chlorophyll *a* or any external particulate input) are a proxy for large-bodied zooplankton, which can be strongly stimulated under eutrophic conditions by the availability of algae (Cole et al., 2000). Despite the source of experimental bias related to any extrapolation from the periphyton response on the microcosm walls to whole ecosystem scale, our results support a potential relevance of N control under P-enriched conditions on algae community. The strength of this zooplankton control on phytoplankton, but not on periphyton biomass in highly nutrient-enriched lake waters was previously reported using experimental mesocosms in another lake at the same studied region as in this work (Guariento et al., 2011). Thus, the absence of common grazers on zooplankton in tropical coastal lagoons, i.e., snails and fishes (Guariento et al., 2010), might have contributed to the increase of the periphyton biomass in clear water nutrient-enriched microcosms.

In conclusion, our hypothesis was confirmed as N is an important driver on *p*CO_2_ in P-enriched coastal lagoons waters. Higher experimental N enrichments promoted a significant *p*CO_2_ decrease in both humic and clear coastal lagoons waters. The N inputs even under P-enriched conditions might lead to intense net decreases in CO_2_ in coastal lagoons waters. Both inorganic N and organic substrates inputs modulate the CO_2_ balance in freshwater and brackish coastal lagoons.

## Conflict of Interest Statement

The authors declare that the research was conducted in the absence of any commercial or financial relationships that could be construed as a potential conflict of interest.
